# Supplementary material for: A Quantitative 1H NMR Method for Screening Cannabinoids in CBD Oils
Source: Toxics. 2021 Jun 10;9(6):136. doi: 10.3390/toxics9060136 (PMC8228318; doi:10.3390/toxics9060136)
Supplement: Supplementary file 1 [file toxics-09-00136-s001.zip › toxics-1241233-supplementary.pdf]

toxics

MDPI

Supplementary Materials: A Quantitative ¹H NMR Method for Screening Cannabinoids in CBD Oils

Ines Barthlott, Andreas Scharinger, Patricia Golombek, Thomas Kuballa and Dirk W. Lachenmeier

Automated quantification routine using PULCON principle

$$f_{ERETIC}\left[\frac{ppm \cdot l}{mol}\right] = \frac{1}{N}\sum_{i}^{N}\frac{(I_{std} \cdot\ k_{sp}) \cdot SW \cdot M_{std}}{SI \cdot \beta_{std} \cdot N^{H}_{std}} \quad \text{(S1)}$$

with
- f_{ERETIC}: device-specific response factor
- I_{std}: absolute integral of the standard signal
- SW: spectral width [ppm]
- M_{std}: molecular weight of the standard [g/mol]
- SI: size of real spectrum
- β_{std}: mass concentration of the standard [mg/l]
- N^{H}_{std}: numbers of protons generating the selected signal
- k_{sp}: spectroscopic correction factor
  $k_{sp}(TCNB) = 1.30$; $k_{sp}(EB) = 1.22$

$$ß_{x}\left[\frac{g}{l}\right] = \frac{I_x \cdot SW \cdot M_x}{SI \cdot f_{ERETIC} \cdot N^{H}_{x} \cdot f_{dil}} \cdot \left(\frac{P1_X}{P1_{Std}} \cdot \frac{NS_{std}}{NS_X}\right) \quad \text{(S2)}$$

with
- β_x: mass concentration of the analyte x [mg/l]
- I_x: absolute integral of the analyte x
- SW: spectral width in [ppm]
- M_x: molecular weight of the analyte x [g/mol]
- SI: size of real spectrum
- f_{ERETIC}: device-specific response factor, see equation (S1)
- N^{H}_{x}: numbers of protons generating the selected signal
- f_{dil}: dilution factor
- P1: pulse of sample (x) or standard (std) [µs]
- NS: number of scans, sample (x) or standard (std)

$$w_x\left[\frac{mg}{kg}\right] = \frac{\beta_x \cdot \left(\frac{W}{\rho} + V_S\right)}{100} \quad \text{(S3)}$$

with
- w_x: mass fraction of cannabinoid content [mg/kg]
- βx: mass concentration of the analyte [mg/l]
- W: weighing [mg]
- ρ: density of CBD oil [g/ml]
- V_S: volume of the solvent [µl]

Table S1. Integration limits used for the quantification of the cannabinoids.

Cannabinoids	Signal Label	Assignment in the Molecule (cf. Figure 1)	Number of Protons	Integration Limit [ppm]
cannabidiol	CBD 1	H-1	1	3.837–3.915
	CBD 2*	H-9 *cis*	1	4.506–4.568
	CBD 3	H-9 *trans*	1	4.597–4.673
cannabinol	CBN 1	H-2	1	7.019–7.074
	CBN 2	H-5	1	7.097–7.153
	CBN 3	H-4	1	8.173–8.250
Δ^9-tetrahydrocannabinol	Δ^9-THC*	H-3′′	1	hemp oil: 6.135–6.148<br>MCT oil: 6.151–6.186
Δ^8-tetrahydrocannabinol	Δ^8-THC*	H-3′′	1	hemp oil: 6.10–6.138<br>MCT oil: 6.1385–6.1524
ethylbenzene	EB	arom. H	3	7.0162–7.2341
tetrachloronitrobenzene	TCNB	arom. H	1	7.64–7.83

* integrated using line fitting algorithm

Composition of standards and solutions in validation studies

Table S2. Mixtures of the standard solutions used for the calibrations.

Standard Solution	Mixture
**cannabidiol standard solution (CBD-S)**	10.130 mg ǀ 5 mL $CDCl_3$
**cannabinol standard solution (CBN-S)**	4.995 mg ǀ 2 mL $CDCl_3$

Table S3. Cannabidiol calibration in the concentration range up to 1 mg/l. CBD-S, cannabidiol standard solution.

Calibration Point	Concentration [mg/L]	Mixture
Blank		103.07 mg hemp seed oil + 600 µl $CDCl_3$
1	114.05	100.18 mg hemp seed oil + 40 µl ml CBD-S + 560 µl $CDCl_3$
2	342.03	100.39 mg hemp seed oil + 120 µl CBD-S + 480 µl $CDCl_3$
3	569.31	101.23 mg hemp seed oil + 200 µl CBD-S + 400 µl $CDCl_3$
4	798.34	100.18 mg hemp seed oil + 280 µl CBD-S + 320 µl $CDCl_3$
5	1024.72	101.26 mg hemp seed oil + 360 µl CBD-S + 240 µl $CDCl_3$

Table S4. Cannabidiol calibration in the concentration range of 7.7–37 g/L.

Calibration Point	Concentration [mg/L]	Mixture
Blank		154.09 mg hemp seed oil ǀ 1 mL
1	7745	7.75 mg CBD + 153.13 mg hemp seed oil ǀ 1 mL $CDCl_3$
2	14970	14.97 mg CBD + 153.73 mg hemp seed oil ǀ 1 mL $CDCl_3$
3	22375	22. 38 mg CBD + 153.91 mg hemp seed oil ǀ 1 mL $CDCl_3$
4	31525	31.53 mg CBD + 155.24 mg hemp seed oil ǀ 1 mL $CDCl_3$
5	36950	36.95 mg CBD + 153.59 mg hemp seed oil ǀ 1 mL $CDCl_3$

Table S5. Cannabinol calibration. CBN-S, cannabinol standard solution.

Calibration Point	Concentration [mg/L]	Mixture
blank		99.90 mg CBD oil (15 %) + 600 µl $CDCl_3$
1	140.36	99.90 mg CBD oil (15 %) + 40 µL CBN-S + 560 µL $CDCl_3$
2	420.71	100.46 mg CBD oil (15 %) + 120 µL CBN-S + 480 µL $CDCl_3$
3	702.23	99.61 mg CBD oil (15 %) + 200 µL CBN-S + 400 µL $CDCl_3$
4	980.38	101.29 mg CBD oil (15 %) + 280 µL CBN-S + 320 µL $CDCl_3$
5	1262.19	100.30 mg CBD oil (15 %) + 360 µL CBN-S + 240 µL $CDCl_3$

*blank = CBD oil sample with declared content 15 % CBD based on hemp seed oil.

Table S6. Δ^9-tetrahydrocannabinol calibration. Sol, solution.

Calibration Point	Concentration [mg/L]	Mixture
blank		101.94 mg CBD oil (10 %)
1	140.29	100 µL Δ^9-THC-Sol + 100.87 mg CBD oil (10 %) + 600 µL $CDCl_3$
2	418.94	300 µL Δ^9-THC-Sol + 103.78 mg CBD oil (10 %) + 600 µL $CDCl_3$
3	700.50	500 µL Δ^9-THC-Sol + 101.71 mg CBD oil (10 %) + 600 µL $CDCl_3$
4	980.83	700 µL Δ^9-THC-Sol + 101.63 mg CBD oil (10 %) + 600 µL $CDCl_3$
5	1259.68	900 µL Δ^9-THC-Sol + 102.34 mg CBD oil (10 %) + 600 µL $CDCl_3$

*blank = self-made CBD oil (10 %) based on hemp seed oil.

Table S7. Δ^8-tetrahydrocannabinol calibration. Sol, solution.

Calibration Point	Concentration [mg/L]	Mixture
blank		100.19 mg CBD oil (10 %)+ 600 µL $CDCl_3$
1	140.33	100 µL Δ^8-THC-Sol + 100.66 mg CBD oil (10 %) + 600 µL $CDCl_3$
2	421.09	300 µL Δ^8-THC-Sol + 100.52 mg CBD oil (10 %) + 600 µL $CDCl_3$
3	697.85	500 µL Δ^8-THC-Sol + 104.14 mg CBD oil (10 %) + 600 µL $CDCl_3$
4	984.23	700 µL Δ^8-THC-Sol + 99.43 mg CBD oil (10 %) + 600 µL $CDCl_3$
5	1259.62	900 µL Δ^8-THC-Sol + 102.37 mg CBD oil (10 %) + 600 µL $CDCl_3$

*blank = self-made CBD oil (10 %) based on hemp seed oil.

Influence of NMR solvents

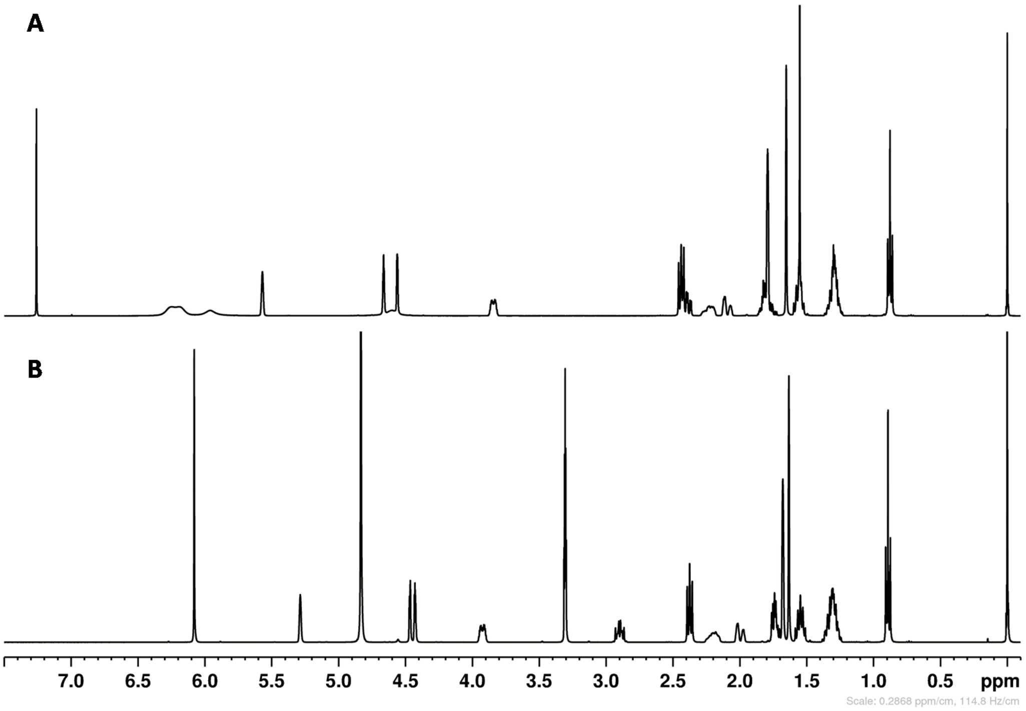

Figure S1. ^{1}H NMR spectra of cannabidiol in (A) deuterated chloroform ($CDCl_3$) and (B) deuterated methanol (CD_3OD).

Signal assignments of the cannabinoids

Table S8. Assignment of the ^{1}H NMR signals of cannabidiol and cannabidiolic acid. With s = singlet, d = doublet, t = triplet, q = quintet, m = multiplet, br = broad.

**Pos.**	**Cannabidiol**		**Cannabidiolic Acid**
	**^{1}H NMR in $CDCl_3$ [a]**	**^{1}H NMR in CD_3OD [a]**	**^{1}H NMR in $CDCl_3$ [a]**
**1**	3.84 (1H, dm, 10.0 Hz)	3.93 (1H, dm, 9.9 Hz)	4.10 (1H, m)
**2**	5.57 (1H, s)	5.29 (1H, s)	5.56 (1H, s)
**3**			
**4**	2.09 (1H, m); 2.24 (1H, m)	1.99 (1H, dd, 9.2 Hz); 2.20 (1H, m)	---
**5**	1.82 (2H, m) [g]	1.74 (2H, m)	---
**6**	2.40 (1H, m) [e]	2.89 (1H, td, 16.2 Hz, 4.5 Hz)	---
**7**	1.79 (3H, s) [g]	1.67 (3H, s)	1.79 (s)
**8**			
**9**	4.67 (*trans*, 1H, m) [c]<br>4.60 (*cis*, 1H, m) [c]	4.47 (*trans*, 1H, m)<br>4.43 (*cis*, 1H, m)	4.54 (*trans*, 1H, m)<br>4.41 (*cis*, 1H, m)
**10**	1.65 (3H, s)	1.63 (3H, s)	1.71 (s)
**1′**			
**2′**			
**3′**	6. 25 (1H, brs) [f]	6.08 (2H, s) [h]	
**4′**			
**5′**	6.19 (1H, brs) [f]	6.08 (2H, s) [h]	6.23 (1H, s)
**6′**			
**1′′**	2.44 (2H, t, 7.6 Hz) [e]	2.38 (2H, t, 7.6 Hz)	2.94 (1H, m), 2.82 (1H, m)
**2′′**	1.56 (2H, q, 15.2 Hz) [b]	1.54 (2H, q, 14.9 Hz)	1.56 (2H, m)
**3′′**	1.30 (4H, m) [d]	1.31 (4H, m) [i]	1.33 (4H, m) [j]
**4′′**	1.30 (4H, m) [d]	1.31 (4H, m) [i]	1.33 (4H, m) [j]
**5′′**	0.89 (3H, t, 6.9 Hz)	0.89 (3H, t, 7.0 Hz)	0.87 (3H, t, 7.0 Hz)
**2′-OH**	5.95 (1H, brs)		9.16 (1H, brs)
**6′-OH**	4.60 (1H, brs)		6.52 (1H, s)
**COOH**			

[a] spectrum recorded with 400 MHz at 300 K, chemical shift [ppm] referred to TMS; [b] signals overlapped with water residues; [c] overlap with OH group; [d-j] signals overlapped in the spectra; --- signal was not assigned.

Table S9. Assignment of the ^{1}H NMR signals of Δ[9]-tetrahydrocannabinol (Δ[9]-THC), Δ[9]-tetrahydrocannabinolic acid A (Δ[9]-THCA-A), Δ[8]-tetrahydrocannabinol (Δ[8]-THC) and Δ[9]-tetrahydrocannabivarin (THCV). With s = singlet, d = doublet, t = triplet, q = quintet, m = multiplet, br = broad.

Pos.	Δ[9]-THC	Δ[9]-THCA A	Δ[8]-THC	THCV
	^{1}H NMR [a]	^{1}H NMR [a]	^{1}H NMR [a]	^{1}H NMR [a]
**1**	3.17 (1H, dm, 11 Hz)	3.23 (1H, dm, 5.6 Hz)	2.70 (1H, td, 11 Hz, 4.8 Hz)	3.20 (1H, m)
**2**	6.30 (1H, q)	6.39 (1H, brs)	3.19 (1H, dd, 16.2 Hz, 3.7 Hz)<br>1.85 (1H, m) [i]	6.30 (1H, q)
**3**				
**4**	2.17 (2H, m)	2.17 (2H, m)	5.43 (1H, brd, 4.7 Hz)	2.17 (2H, m)
**5**	1.91 (1H, m)<br>1.40 (1H, m) [c]	1.92 (1H, m)<br>1.43 (1H, m) [f]	2.14 (1H, m)<br>1.81 (1H, m) [i]	1.91 (1H, m)<br>1.40 (1H, m) [k]
**6**	1.70 (1H, m) [d]	1.69 (m) [g]	1.79 (1H, m) [i]	1.69 (1H, m) [l]
**7**	1.69 (3H, s) [d]	1.68 (3H, s) [g]	1.70 (3H, s)	1.67 (3H, m) [l]
**8**				
**9**	1.40 (3H, s) [c]	1.44 (3H, s) [f]	1.37 (3H, s)	1.40 (3H, s) [k]
**10**	1.09 (3H, s)	1.11 (3H, s)	1.10 (3H, s)	1.09 (3H, s)
**1′**				
**2′**				
**3′**	6.14 (1H, d, 1.6 Hz)		6.10 (1H, d, 1.6 Hz)	6.14 (1H, d, 1.6 Hz)
**4′**				
**5′**	6.26 (1H, d, 1.5 Hz)	6.25 (1H, s)	6.27 (1H, d, 1.5 Hz)	6.26 (1H, d, 1.6 Hz)
**6′**				
**1′′**	2.44 (2H, td, 7.6 Hz, 1.6 Hz)	2.94 (1H, m)<br>2.77 (1H, m)	2.44 (2H, td, 7.9 Hz, 3.1 Hz)	2.42 (2H, td, 7.5 Hz, 2.2 Hz)
**2′′**	1.55 (q) [b]	1.57 (m) [b]	1.56 (m) [b]	1.57 (m) [b]
**3′′**	1.30 (4H, m) [e]	1.31 (4H, m) [h]	1.30 (4H, m) [j]	0.91 (3H, t, 7.3 Hz)
**4′′**	1.30 (4H, m) [e]	1.31 (4H, m) [h]	1.30 (4H, m) [j]	
**5′′**	0.88 (3H, t, 7.0 Hz)	0.90 (3H, t, 7.0 Hz)	0.88 (1H, t,7.1 Hz)	
**2′-OH**	4.69 (1H, s)	12.23 (1H, s)	4.62 (1H, s)	4.70 (1H, s)
**COOH**				

[a] spectrum recorded with 400 MHz at 300 K, chemical shift [ppm] referred to TMS; [b] signals overlapped with water residues; [f - l] signals overlapped in the ^{1}H NMR spectra.

Table S10. Assignment of the ^{1}H NMR signals of cannabigerol and cannabinol. With s = singlet, d = doublet, t = triplet, q = quintet, m = multiplet, br = broad.

Pos.	Cannabigerol	Pos.	Cannabinol
	^{1}H NMR [a]		^{1}H NMR [a]
1		1	
2		2	8.15 (1H, s)
3		3	
4	6.25 (2H, s) [c]	4	7.07 (1H, d, 8.0 Hz)
5		5	7.14 (1H, d, 7.8 Hz)
6	6.25 (2H, s) [c]	6	
1′	3.39 (2H, d, 7.1 Hz)	7	2.38 (3H, s)
2′	5.27 (1H, m)	8	
3′		9	1.59 (s) [b, g]
3′-Me	1.81 (3H, s)	10	1.59 (s) [b, g]
4′	2.08 (4H, m) [d]	1′	
5′	2.08 (4H, m) [d]	2′	
6′	5.05 (1H, m)	3′	6.30 (1H, d, 1.5 Hz)
7′		4′	
8′	1.59 (s) [b, e]	5′	6.44 (1H, d, 1.5 Hz)
9′	1.67 (3H, s)	6′	
1′′	2.46 (2H, t, 7.6 Hz)	1′′	2.50 (2H, t, 7.8 Hz)
2′′	1.56 (q) [b, e]	2′′	1.61 (m) [b, g]
3′′	1.31 (4H, m) [f]	3′′	1.32 (4H, m) [h]
4′′	1.31 (4H, m) [f]	4′′	1.32 (4H, m) [h]
5′′	0.88 (3H, t, 6.8 Hz)	5′′	0.89 (3H, t, 7.2 Hz)
OH	4.96 (2H, s)	2′-OH	5.12 (1H, s)

[a] spectrum recorded with 400 MHz at 300 K, chemical shift [ppm] referred to TMS; [b] signals overlapped with water residues; [c–h] signals overlapped in the ^{1}H NMR spectra.

Optimization of NMR protocol receiver gain and signal to noise ratio

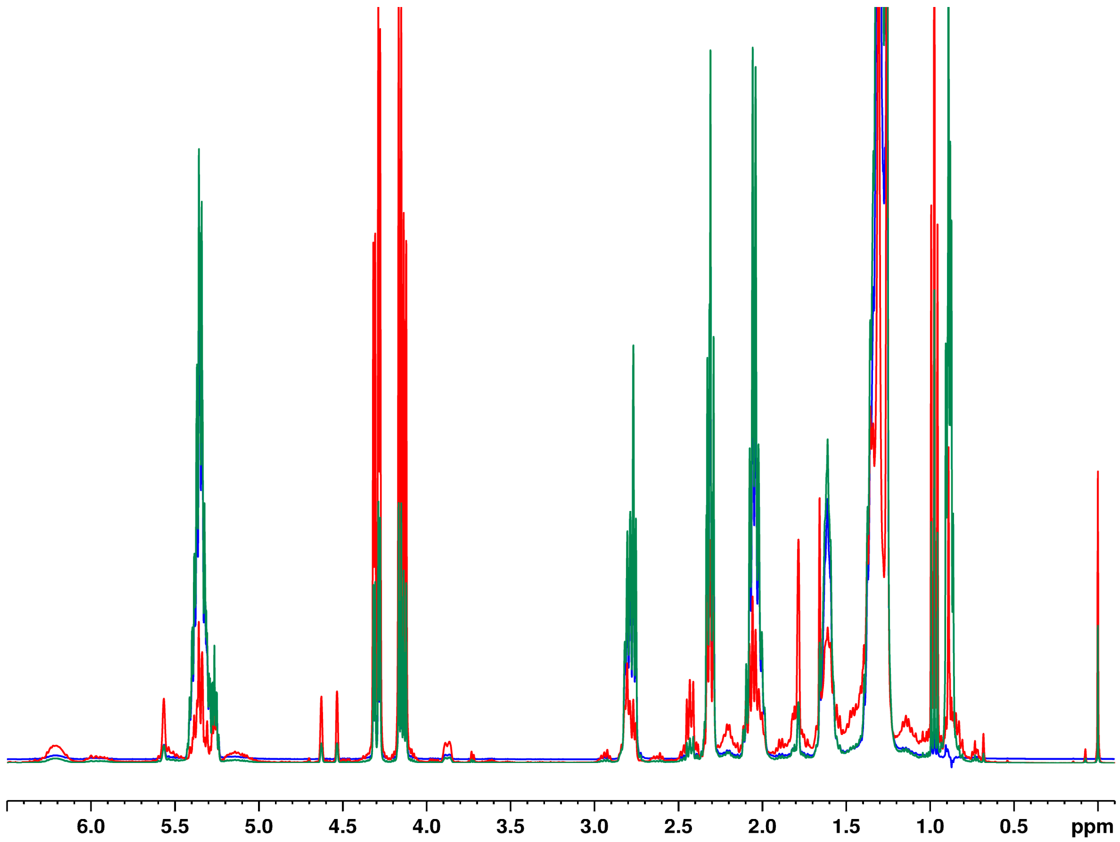

Figure S2. ^1H NMR spectra of a CBD oil in $CDCl_3$ recorded with different suppression programs. Blue, with suppression of the CH_3 lipid signal (RG: 8); Green, without suppression (RG: 5.6); Red, multiple suppression (RG: 16). The results were obtained with a sample solution of 100 mg oil dissolved in 600 µL $CDCl_3$.

Method validation: linearity

Table S11. Calibration data of the investigated cannabinoid signals. CBD, cannabidiol; Δ9-THC, Δ9-tetrahydrocannabinol; CBN, cannabinol; Δ8-THC, Δ8-tetrahydrocannabinol; THCA Δ9-tetrahydrocannabinolic acid A; c, concentration; δ, chemical shift; H signal, proton signal; R, correlation coefficient; s_{x0}, process standard deviation; v_{x0}, coefficient of variation.

Cannabinoid	C [mg/L]	H Signal	δ [ppm]	R	s_{x0} [mg/L]	v_{x0} [%]
**CBD**	114.05–1024.72	H-1	3.837–3.915	0.99932	15.34	2.69
		H-9 *cis*	4.506–4.568	0.9999	5.93	1.04
		H-9 *trans*	4.597–4.673	0.99946	13.62	2.39
**CBD**	7745–36950	H-1	3.837–3.915	0.99981	269.74	1.21
		H-9 *cis*	4.506–4.568	0.99985	237.52	1.06
		H-9 *trans*	4.597–4.673	0.99998	87.28	0.39
**Δ9-THC**	140.29–1259.68	H-3′	6.135–6.148	0.99862	26.92	3.85
**CBN**	140.36–1262.45	H-2	7.019–7.074	0.999	22.88	3.26
		H-5	7.097–7.153	0.99856	27.54	3.93
		H-4	8.173–8.250	0.99905	22.32	3.18
**Δ8-THC**	140.33–1259.62	H-3′	6.10–6.138	0.99977	11.08	1.58

Method validation: recovery

Table S12. Recoveries of the cannabinoids in [%]. CP, calibration point; CBD, cannabidiol; CBN, cannabinol; Δ9-THC, Δ9-tetrahydrocannabinol; Δ8-THC, Δ8-tetrahydrocannabinol.

Signal	CBD 1*	CBD 2*	CBD 3*	CBN 1	CBN 2	CBN 3	Δ8-THC	Δ9-THC
**CP 1**	120	117	117	117	125	120	57	35
**CP 2**	122	118	117	119	123	123	86	62
**CP 3**	119	114	115	119	122	124	93	80
**CP 4**	119	115	114	126	131	132	92	84
**CP 5**	117	113	114	129	135	134	93	84

*recovery of the calibration in the high concentration range; dark gray = concentration of the CP under the LOD; light gray = concentration of the CP under the LOQ.

Table S13. Recovery in [%] of cannabidiol (CBD) determined from calibration without matrix. CP, calibration point.

signal*	CBD 1 (H-1)	CBD 2 (H-9 *cis*)	CBD 3 (H-9 *trans*)	H-2	H-3′′ + 5′′	H-5′′	H-10	H-1′′ + 6
**CP 1**	128	133	77	121	124	11	114	124
**CP 2**	122	129	79	117	122	14	106	119
**CP 3**	122	128	79	117	122	10	107	119
**CP 4**	123	129	79	117	122	10	106	119
**CP 5**	121	127	79	117	124	10	104	117

* assignment of the cannabidiol signals according to Figure 1; dark gray = signals overlapped with OH-group; light gray = signal influenced by multiple suppression.

Comparison and fit of NMR and LC-MS/MS results for Δ⁹-THC

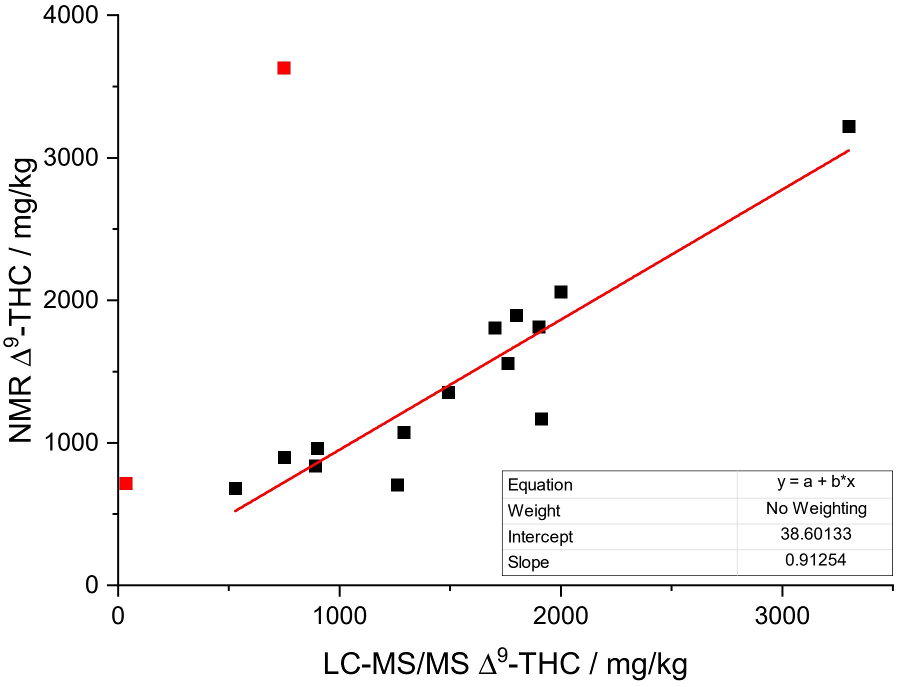

Figure S3. Linear fit of NMR and LC-MS/MS results of all samples with NMR results above the NMR(LOD).
